# The domestication of the wolf larynx—testing the neural crest connection

**DOI:** 10.1098/rsos.250430

**Published:** 2025-07-09

**Authors:** Jacob Hansen, Nicole D. S. Grunstra, W. Tecumseh Fitch, Andrew C. Kitchener, Michaela Gumpenberger, Raffaela Lesch

**Affiliations:** ^1^ Department of Biology, University of Arkansas at Little Rock, Little Rock, AR, USA; ^2^ Department of Evolutionary Biology, University of Vienna, Vienna, Austria; ^3^ Natural History Museum Vienna, Vienna, Austria; ^4^ Department of Behavioral and Cognitive Biology, University of Vienna, Vienna, Austria; ^5^ Department of Natural Sciences, National Museums Scotland, Edinburgh, UK; ^6^ Clinic of Diagnostic Imaging, University of Veterinary Medicine Vienna, Vienna, Austria

**Keywords:** domestication, wolf, larynx, neural crest, NCDS

## Abstract

The neural crest domestication syndrome (NCDS) hypothesis suggests that selection pressures for tameness during animal domestication resulted in a down-regulation of neural crest cell migration and proliferation, providing a mechanistic explanation for traits commonly observed across domesticates. We test the viability of this hypothesis via a derived prediction. According to the NCDS hypothesis, neural crest-derived structures should be reduced throughout the entire organism in domesticated variants compared to their wild ancestors. Here, we test this prediction by comparing the larynges of wolves and domestic dogs. Anatomical landmarking of the cricoid and thyroid cartilages confirmed absolutely and relatively shorter vocal folds and smaller larynges in normal (mesocephalic) dogs compared to wolves. Additional quantification of laryngeal anatomy shows that mesocephalic dogs have a smaller cricoid diameter and shape-altered thyroid shields. In addition, we quantify the concrete anatomical changes to the laryngeal cartilages caused by brachycephaly, finding that a flattened thyroid shield and ventral cricoid ring fit the symptomatic descriptions of laryngeal collapse. Our comparison of the larynges of wolves and dogs are consistent with the NCDS-derived prediction and support the validity of the NCDS hypothesis.

## 1. Introduction

Animal domestication has played a crucial role in the formation of human societies and helped jump-start the Neolithic Revolution, when there was a profound shift from hunter–gatherer communities to settlements dominated by agriculture. This shift occurred due to domestication of wild animals and plants in small communities to produce crops and livestock [1]. Domestication of animals in its initial stages was a process of natural selection on wild animals that were trying to exploit human habitats and favoured individual animals that were best able to tolerate the proximity of humans [2,3]. The process of domestication is a change along a continuum, moving from the initial natural selection pressures to active selection of particular traits by humans [4]. The movement along this continuum from wild to domesticate is associated with changes called the ‘domestication syndrome’ [5].

The domestication syndrome describes traits seen across certain varieties of all domesticated mammal species, such as floppy ears, curly tails, piebald patterns (referred to as depigmentation), smaller brains and reduced snouts. These traits often occur in combination with each other, though there is variability in occurrence and connected patterns across taxa [6]. These common traits received a potential developmental explanation through the neural crest domestication syndrome (NCDS) hypothesis [7,8]. The NCDS hypothesis suggests that close proximity to humans associated with active and passive selection pressures for tameness (the ultimate necessity for coexistence with humans) have resulted in a down-regulation of neural crest cell migration and proliferation [7,8]. Neural crest cells, through their migratory pattern, provide progenitor cells for various systems during mammalian development, explaining all traits readily captured within the domestication syndrome [9]. This hypothesis is the most popular and widely accepted explanation underlying the domestication phenotype, but remains yet to be tested comprehensively, as pointed out by critics [10–12]. Common criticisms regarding the NCDS apply to both the validity of the domestication syndrome as a ubiquitous phenotype and the validity of the mechanistic explanation underlying the NCDS hypothesis [12–15]. Here, we directly address the validity of the mechanistic pathway suggested by the hypothesis.

The NCDS hypothesis lays out readily testable predictions. Domestic animals should have a slight deficiency in the migration and proliferation of neural crest cells, which in turn should lead to fewer progenitor cells arriving at target sites during embryogenesis; any structure connected to the neural crest cells should therefore be ‘reduced’ in domesticates compared to their wild ancestors. Wilkins *et al*. [7] provide an overview of organ systems linked to the neural crest with clear pathways for morphological changes (such as snout reduction) and suggest others of more speculative nature. Lesch & Fitch [16] specifically outline a connection between the development of laryngeal structures and neural crest cells. The larynx is an organ of dual function, protecting the airways from inhalation of food and water, as well as being the source of mammalian voice production [17]. The mammalian larynx is a mostly cartilaginous (varyingly mineralized) structure, composed of a ring-shaped cricoid (below which the airways continue to the lungs), a shield-shaped thyroid, paired arytenoids, vocal folds, the epiglottic cartilage and the hyoid bones (which connect the entire laryngeal structure to both the tongue and skull [18]). The vocal folds, spanning between the thyroid and arytenoid cartilages, provide the source of the mammalian voice. Air passing through the larynx during vocal fold adduction results in vocal fold vibration. Recent cell lineage tracing studies connect both the vocal folds as well as the entire thyroid cartilage to neural-crest-cell-derived origins [19–21]. Hence, a reduction in neural crest cells, induced by domestication, should directly impact both the thyroid cartilage and vocal folds. Lesch & Fitch [16] predicted a smaller and differently shaped larynx in domesticates. Here, we set out to test that prediction in a sample of dogs and wolves, thereby addressing the mechanistic pathway suggested in the NCDS hypothesis.

Aside from the main focus of testing NCDS-derived predictions of laryngeal anatomy, we also explore exact morphological changes in a subpopulation of dogs suffering from the brachycephalic airway syndrome (BAS). BAS is a well-known cause of airway deformities in domestic dogs with massively reduced snouts (‘brachycephaly’ [22]). Aside from constrictions of the anterior airways, the tip of the soft palate often extends over the epiglottis, causing issues in both protection of the larynx and free movement of air through the larynx [23]. Everted laryngeal saccules (extensions between the vocal and ventricular folds) further narrow the airways and affect opening of the glottis [24]. These specific and well-described functional impairments to the airways make this particular group ideal in providing a ceiling of extreme anatomical changes in response to severe artificial selection in subpopulations. Therefore, we distinguish between mesocephalic (normal skull anatomy) and brachycephalic dog breeds in our analyses, since the NCDS makes predictions concerning early domestication, but differences between brachycephalic dogs and wolves may be artefacts of relatively recent breed selection.

The comparison of laryngeal anatomy across domestic dog breeds and their extant wild counterparts (i.e. wolves) will yield a conclusive test of the validity of the NCDS hypothesis as applied to laryngeal morphology.

## 2. Methods

### 2.1. Specimens

We collected scans of a total of nine wolf (*Canis lupus*) and 25 domestic dog (*C. familiaris*) larynges (table 1). Two wolf and four dog larynx scans were excluded from the analysis due to insufficient scan quality. Of the remaining samples four of the wolf larynges originated from individuals donated to National Museums of Scotland, Edinburgh, UK. These were excised on-site before transportation to Vienna, Austria. The other three wolf larynges originated from CT scans of live wolves kept at the Wolf Science Center Ernstbrunn and scanned for medical reasons at the University of Veterinary Medicine in Vienna, Austria. All CT scans from 21 dogs were sampled from patients at the University of Veterinary Medicine. Eight of the dog specimens were brachycephalic and 13 were mesocephalic.

**Table 1.** Table of all individuals and scans available to this study, including species, snout length, origin, breed (if applicable) and type of scan (CT scan of live animal or excised larynx). Individuals excluded from the analysis are marked via the ID ending “_excl”.

ID	species	nose	origin	breed	vocalization	scan
CANLUP001_excl	*C. lupus*	mesocephalic	Scotland	—	howl	excised
CANLUP002	*C. lupus*	mesocephalic	Scotland	—	howl	excised
CANLUP003_excl	*C. lupus*	mesocephalic	Scotland	—	howl	excised
CANLUP004	*C. lupus*	mesocephalic	Scotland	—	howl	excised
CANLUP005	*C. lupus*	mesocephalic	Scotland	—	howl	excised
CANLUP006	*C. lupus*	mesocephalic	Scotland	—	howl	excised
T2009_9695	*C. lupus*	mesocephalic	Vienna	—	howl	live
T2012_4506	*C. lupus*	mesocephalic	Vienna	—	howl	live
T2021_4741	*C. lupus*	mesocephalic	Vienna	—	howl	live
HUND1_excl	*C. familiaris*	mesocephalic	Vienna	na	bark	live
HUND2_excl	*C. familiaris*	mesocephalic	Vienna	na	bark	live
HUND3	*C. familiaris*	mesocephalic	Vienna	na	bark	live
HUND4	*C. familiaris*	mesocephalic	Vienna	na	bark	live
HUND5_excl	*C. familiaris*	mesocephalic	Vienna	na	bark	live
HUND6	*C. familiaris*	mesocephalic	Vienna	na	bark	live
HUSKY1	*C. familiaris*	mesocephalic	Vienna	Husky	howl	live
HUSKY2	*C. familiaris*	mesocephalic	Vienna	Husky	howl	live
HUSKY3_excl	*C. familiaris*	mesocephalic	Vienna	Husky	howl	live
HUSKY4	*C. familiaris*	mesocephalic	Vienna	Husky	howl	live
T2015_2105	*C. familiaris*	brachycephalic	Vienna	French bulldog	bark	live
T2017_13447	*C. familiaris*	brachycephalic	Vienna	French bulldog	bark	live
T2018_1448	*C. familiaris*	brachycephalic	Vienna	French bulldog	bark	live
T2020_5988	*C. familiaris*	brachycephalic	Vienna	French bulldog	bark	live
T2021_2744	*C. familiaris*	brachycephalic	Vienna	French bulldog	bark	live
T2021_4128	*C. familiaris*	brachycephalic	Vienna	French bulldog	bark	live
T2021_7540	*C. familiaris*	mesocephalic	Vienna	German shorthaired pointer	bark	live
T2021_7605	*C. familiaris*	mesocephalic	Vienna	Border collie	bark	live
T2021_7704	*C. familiaris*	brachycephalic	Vienna	French bulldog	bark	live
T2021_8148	*C. familiaris*	mesocephalic	Vienna	German shepherd	bark	live
T2021_8417	*C. familiaris*	mesocephalic	Vienna	Beauceron	bark	live
T2021_11029	*C. familiaris*	mesocephalic	Vienna	Border collie	bark	live
T2021_11297	*C. familiaris*	mesocephalic	Vienna	na	bark	live
T2022_138	*C. familiaris*	brachycephalic	Vienna	French bulldog	bark	live
T2022_386	*C. familiaris*	mesocephalic	Vienna	Bernese mountain dog	bark	live

### 2.2. Relevant parameters and variables

The main goal of this study is to test the predictions of the NCDS hypothesis and quantify changes to laryngeal size and shape, including relative vocal fold length associated with the process of domestication. To successfully disentangle effects of domestication, artificial selection, scan type (whole body versus excised larynx), snout length, and main vocalisation strategy, we included the following variables in our analysis and models: scan type (excised, live), vocal type (howling—huskies and wolves, barking—all other dogs) and our populations of wolves and dogs (wolves, mesocephalic dogs, brachycephalic dogs).

### 2.3. 3D reconstruction

**Figure 1.**
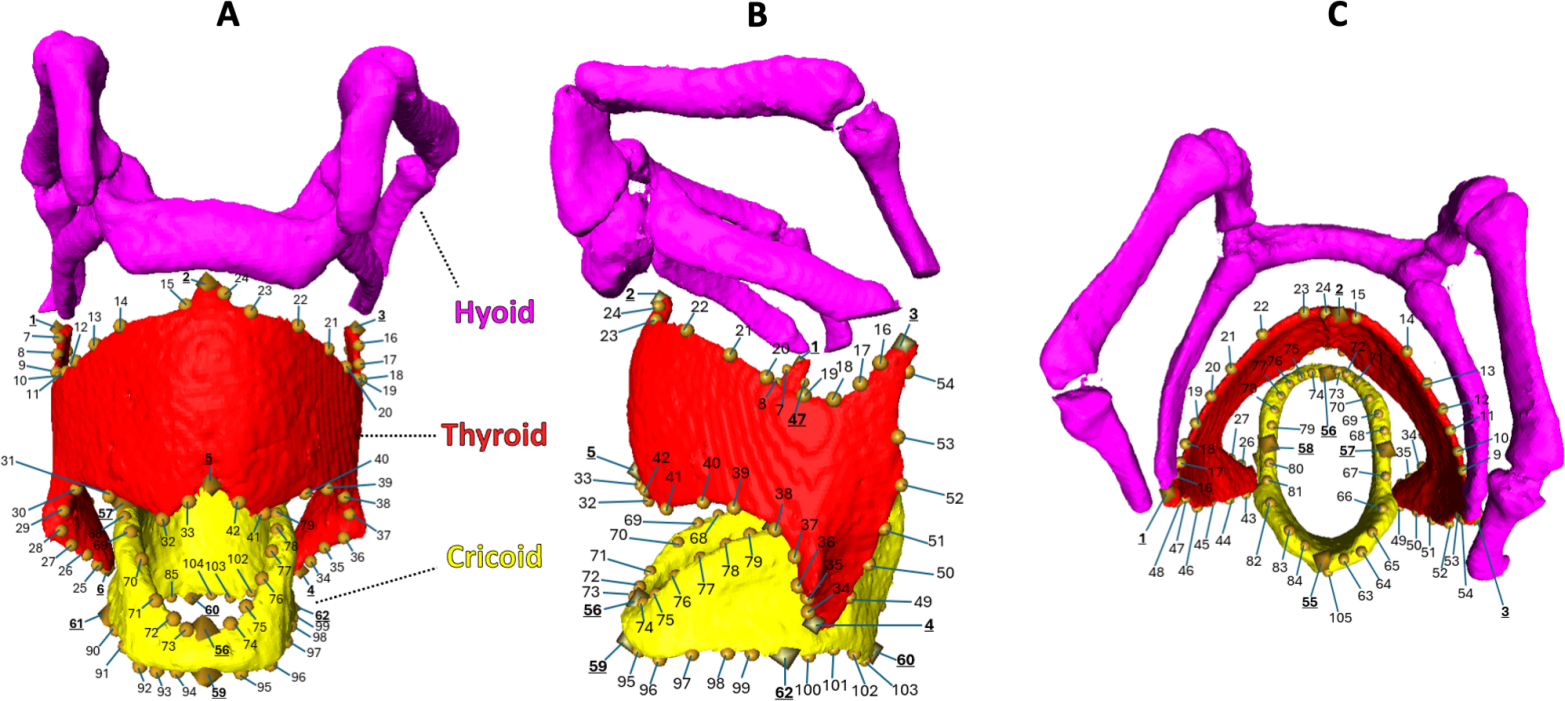
Three-dimensional reconstruction of a *Canis lupus* larynx including landmark positions. Fixed landmarks are indicated by a bold, underlined font and diamond-shaped points, semi-landmarks are indicated by rounded points. The landmark numbers are based on the order of placement. The hyoid is coloured pink, the thyroid red and the cricoid yellow. (A) Ventral, (B) lateral and (C) cranial reconstructions.

After specimen collection the CT image stacks (DICOM files) were uploaded to the Thermo Fisher image data analysis programme Amira-Avizo (version 2022.2 4.6.0.). All scans were segmented manually using the segmentation editor and brush tool. The thyroid cartilage, cricoid cartilage, hyoid bone and skull were segmented into individual materials to guarantee accurate reconstruction of individual structures (figure 1). While all cartilages and the hyoid bones were reconstructed, we limited our analysis to the thyroid and cricoid cartilages. The arytenoid cartilages were too small to offer reliable landmarks across scans. The hyoid bones were excluded due to the nature of some scans being derived from live and others from excised larynges, changing the orientation of the bones.

### 2.4. Landmarking

After completion of the three-dimensional reconstruction, homologous landmarks were placed on the surfaces of the reconstructions. We used anatomical and semi-landmarks to capture laryngeal cartilage shape based on the landmark placement from Borgard *et al*. [25]. Anatomical or fixed landmarks used in this study are defined as points of maximum curvature, such as the maximum curvature of the cranial thyroid horn (also known as type II landmarks as per [26]). We also used semi-landmarks, also known as sliding landmarks and a modification of the original type III [26], for the application to three-dimensional data [27], to capture homologous curves such as the cranial cricoid rim. Each individual’s laryngeal anatomy was captured by 108 landmarks; six anatomical landmarks and 48 semi-landmarks were recorded from the thyroid cartilage, and eight anatomical and 46 semi-landmarks were recorded from the cricoid (table 2).

**Table 2.** Descriptions of the landmark locations on the thyroid and cricoid cartilages. The fixed landmarks (1-6 and 56-62) are placed on the thyroid and cricoid, respectively. The semi-landmarks are placed along the curves between each fixed landmark. The landmark number indicates the placement position of each individual landmark, ‘location’ denotes thyroid and cricoid cartilages, and landmark ‘type’ indicates whether the landmark is a fixed or semi-landmark.

ID number	location	landmark	description
1	thyroid	fixed	posterior point on rostral margin of left cranial process
2	thyroid	fixed	midline point on cranial margin
3	thyroid	fixed	posterior point on cranial margin of right cranial process
4	thyroid	fixed	posterior-most point on caudal margin of right caudal process
5	thyroid	fixed	midline point on caudal margin
6	thyroid	fixed	posterior-most point on caudal margin of left caudal process
7−15	thyroid	semi	curve between posterior point on rostral margin of left cranial process and midline point on cranial margin (between landmarks 1 and 2)
16−24	thyroid	semi	between midline point on cranial margin and posterior point on cranial margin of right cranial process (landmarks 2 and 3)
34−42	thyroid	semi	between posterior-most point on caudal margin of right caudal process and midline point on caudal margin (landmarks 4 and 5)
43−48	thyroid	semi	between posterior point on rostral margin of left cranial process and posterior-most point on caudal margin of left caudal process (landmarks 1 and 6)
49−54	thyroid	semi	between posterior point on cranial margin of right cranial process and posterior-most point on caudal margin of right caudal process (landmarks 3 and 4)
55	cricoid	fixed	posterior-most point on cranial margin
56	cricoid	fixed	anterior-most point on cranial margin
57	cricoid	fixed	right-most point on cranial margin
58	cricoid	fixed	left-most point on cranial margin
59	cricoid	fixed	anterior-most point on caudal margin
60	cricoid	fixed	posterior-most point on caudal margin
61	cricoid	fixed	left-most point on caudal margin
62	cricoid	fixed	right-most point on caudal margin
63−67	cricoid	semi	posterior-most point on cranial margin and right-most point on cranial margin (landmarks 55 and 57)
68−73	cricoid	semi	between anterior-most point on cranial margin and right-most point on cranial margin (landmarks 56 and 57)
74−79	cricoid	semi	between anterior-most point on cranial margin and left-most point on cranial margin (landmarks 56 and 58)
80−84	cricoid	semi	between posterior-most point on cranial margin and left-most point on cranial margin (landmarks 55 and 58)
85−89	cricoid	semi	between posterior-most point on caudal margin and left-most point on caudal margin (landmarks 60 and 61)
90−94	cricoid	semi	between anterior-most point on caudal margin and left-most point on caudal margin (landmarks 69 and 61)
95−99	cricoid	semi	between anterior-most point on caudal margin and right-most point on caudal margin (landmarks 59 and 62)
100−104	cricoid	semi	between posterior-most point on caudal margin and right-most point on caudal margin (landmarks 60 and 62)
105−108	cricoid	semi	between posterior-most point on cranial margin and posterior-most point on caudal margin (landmarks 55 and 60)

### 2.5. Statistical analysis

Geometric morphometric analysis was performed using the packages geomorph (4.0.6 [28]), and Morpho (2.12 [29]), via R (4.3.1) and RStudio (2023.12.1). After successful completion of the three-dimensional model reconstruction and landmark placement, all links, i.e. landmark neighbours, were defined and combined into curves connecting all fixed landmarks of the thyroid and cricoid cartilages via semi-landmarks. Prior to any analyses, we visually confirmed the correct placement and order of all landmarks. After completion of the visual inspection, we symmetrized our landmark data, using the function ‘symmetrize()’ [Morpho], to reduce noise introduced by the segmentation or slight deformations (e.g. caused by pressure on the neck) unrelated to the actual anatomy. We defined the axis of symmetrisation as the sagittal plane in the midline via the corresponding landmarks. Mirrored partners across the sagittal plane were averaged in their position.

We conducted a generalized Procrustes analysis (GPA), which standardizes for variation in scale, overall position, and orientation, via the ‘procSym()’ function [Morpho], choosing step size 1 for semi-landmark sliding. Afterwards, we visualized every landmark set. When checking for outliers, we found three individuals above the upper quartile: T2015_2105, 2021_7704 and T2017_13447. Visual inspection confirmed that the deformed nature of these brachycephalic individuals' larynges due to the brachycephalic airway syndrome (BAS) was the underlying cause of them being flagged as outliers.

We calculated the vocal fold length by measuring the distance between landmarks 5 and 55; landmark 5 sits on the central caudal edge of the thyroid cartilage, and landmark 55 on the sagittal mid-section point at the very top of the cricoid (figure 1). Absolute vocal fold length was calculated as interlandmark distance prior to the GPA, whereas relative vocal fold length was established by creating a ratio of absolute length divided by centroid size. All principal components were tested for allometric relationships with the centroid size of the landmark configuration (i.e. overall laryngeal size).

We ran linear models on overall laryngeal shape (i.e. collection of all landmarks) and size (i.e. centroid size) via the function ‘procD.lm’ and used regular linear models (lm) for both absolute and relative vocal fold length (length relative to larynx centroid size). All full models were compared to their corresponding null models. The full model included vocal production type (howl or bark), scan type (live or excised) and population identity (wolf, mesocephalic dog and brachycephalic dog) as predictors in each full model, i.e. response variable~population + vocalization + scan. The null model only included the average of the data distribution as a predictor. Only full models significantly better than the null models were further analysed for significance of individual variables. After running the models on the complete dataset, we reran all models on a reduced dataset (*n* = 20), only comparing the influence of wolves and mesocephalic dogs on the shape and size of the larynx to rule out result distortion via the extreme anatomy of the brachycephalic dogs.

## 3. Results

### 3.1. Larynx size and vocal fold length

**Figure 2.**
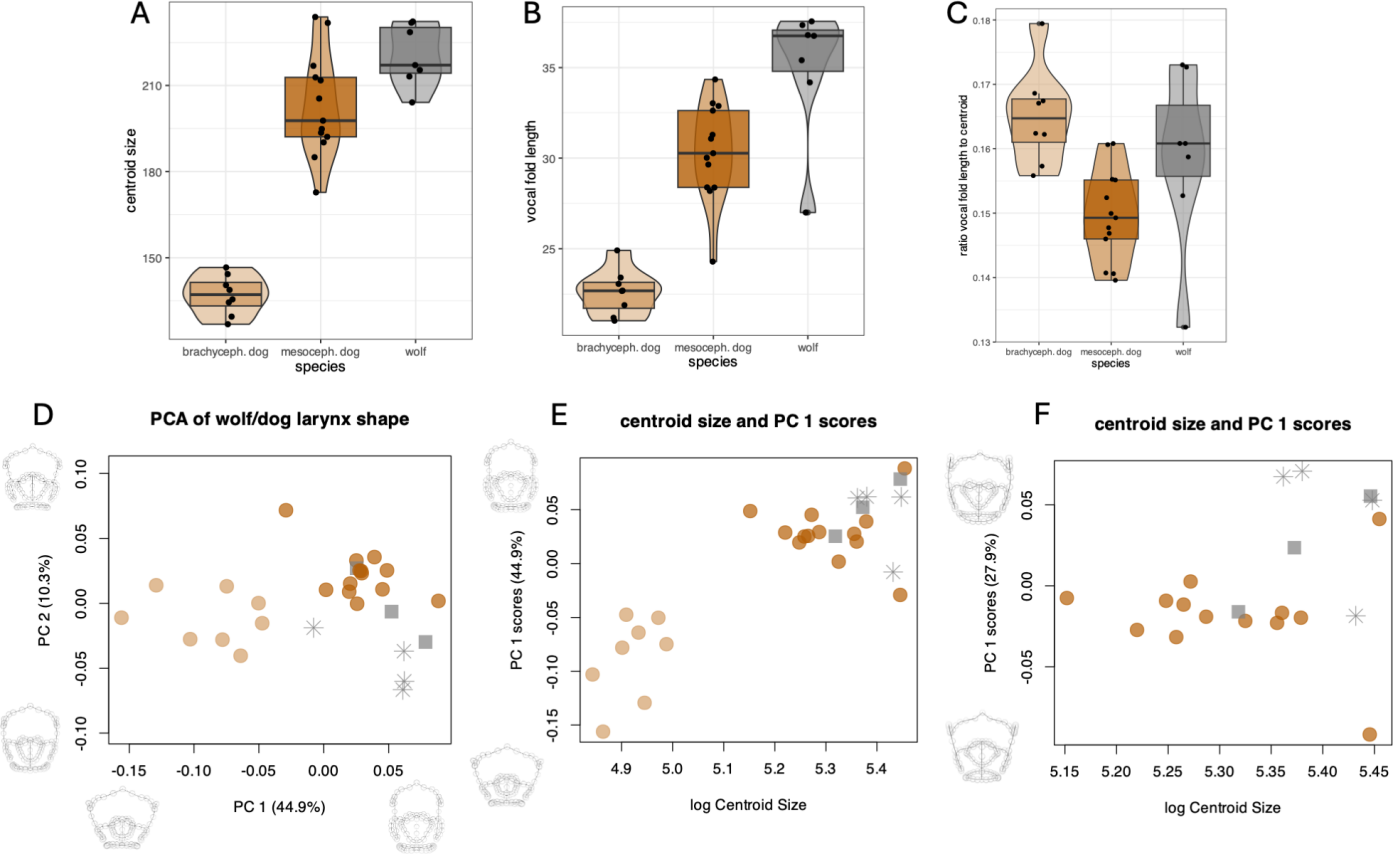
Overview of changes to laryngeal morphology across sample groups. Brachycephalic dogs are illustrated in light brown, mesocephalic dogs in dark brown and wolves in grey (excised larynx as an asterisk, live individual as square). (A) Centroid size by sample group. (B) Absolute vocal fold length by sample group. (C) Vocal fold length relative to the centroid size by sample group. (D) Scores of PC 2 (domestic compression) plotted against PC 1 (BAS flattening). The laryngeal shape changes due to BAS flattening and domestic compression are illustrated along the *x* and *y* axes. (E) Scores of PC 1 (BAS flattening) plotted against logarithmic laryngeal centroid size. The laryngeal shape changes due to BAS flattening are illustrated along the *y* axis. (F) Scores of PC 1 of the reduced dataset, excluding brachycephalic dogs, plotted against centroid size. PC 1 here is similar to the domestic shape change indicated by PC 2 of the full dataset.

The full-null model comparisons for larynx size, as well as absolute and relative vocal fold length, indicated the full models to be significantly better than their respective null models (table 3). Population identity had a significant effect on both larynx size, represented by the centroid size calculated across all landmarks, and on vocal fold length. Brachycephalic dog larynges were the smallest, mesocephalic dog larynges were larger than brachycephalic dogs’, yet smaller than wolf larynges (wolf mean: 220.35, mesocephalic dog mean: 202.96, brachycephalic dog mean: 137.09; figure 2A). Consistent with this difference in overall larynx size, absolute vocal fold length was the shortest in brachycephalic dogs and the longest in wolves. (figure 2B). The derived measure, relative vocal fold length, was also longer in wolves compared to mesocephalic dogs. However, brachycephalic dogs had the longest vocal folds relative to larynx size (wolf mean: 0.1587, mesocephalic dog mean: 0.1496, brachycephalic dog mean: 0.165; figure 2C). The high vocal fold to centroid size ratio of brachycephalic dogs is most likely associated with the severely dysfunctional laryngeal anatomy discussed in more detail in the discussion.

**Table 3.** Overview of the model summaries for centroid size and shape from the function ‘procD.lm’ (which performs a Procrustes ANOVA with permutation procedures) and absolute and relative vocal fold length from a linear model.

centroid size model							
log(centroid size)~population+vocalization+scan							
	Pr(>*F*)						
full-null comparison	0.001						
	d.f.	sum of squares	mean square	*R* ^2^	*F*	*Z*	Pr(>*F*)
population	2	1.03861	0.51931	0.89271	97.5206	6.1579	0.001
vocalization type	1	0.00114	0.00114	0.00098	0.2138	−0.3603	0.641
scan type	1	0.00121	0.00121	0.00104	0.2269	−0.3486	0.628
residuals	23	0.12248	0.00533	0.10527			
total	27	1.16344					

shape model							
coordinates~population+vocalization+scan							
	Pr(>*F*)						
full-null comparison	0.001						
	d.f.	sum of squares	mean square	*R* ^2^	*F*	*Z*	Pr(>*F*)
population	2	0.106731	0.053366	0.42598	9.6231	3.6259	0.001
vocalization type	1	0.00692	0.00692	0.02762	1.2478	0.7986	0.203
scan type	1	0.009358	0.009358	0.03735	1.6874	1.47	0.068
residuals	23	0.127548	0.005546	0.50906			
total	27	0.250556					

vocal fold length (absolute)				
vocal fold length~population+vocalization+scan				
	Pr(>*F*)			
full-null comparison	2.24 × 10^-8^			
	estimate	s.e.	*t*-value	Pr(>|*t*|)
intercept*	27.5292	2.0582	13.375	2.46 × 10^−12^
population—meso. dog	7.5626	1.16	6.519	1.19 × 10^−6^
population—wolf	8.8628	2.3093	3.838	0.000841
vocalization—howl	0.7256	1.6099	0.451	0.656425
scan—live	−4.9213	1.8678	−2.635	0.014808
*intercept includes population—brachycephalic dogs, vocalization—barks and scans—excised residual s.e.: 2.446 on 23 d.f.; Adjusted *R*^2^: 0.7932 *F*-statistic: 26.88 on 4 and 23 d.f.				

vocal fold length (relative to centroid size)				
vocal fold length~population+vocalization+scan				
	Pr(>*F*)			
full-null comparison	0.0003056			
	estimate	s.e.	*t*-value	Pr(>|*t*|)
intercept*	0.183933	0.006549	28.084	<2 × 10^−16^
population—meso. dog	−0.01712	0.003691	−4.638	0.000115
population—wolf	−0.0245	0.007348	−3.334	0.002883
vocalization—howl	0.007386	0.005123	1.442	0.162818
scan—live	−0.018912	0.005943	−3.182	0.004154
*intercept includes population—brachycephalic dogs, vocalization—barks and scans—excised residual s.e.: 0.007782 on 23 d.f.; Adjusted *R*^2^: 0.5139 *F*-statistic: 8.136 on 4 and 23 d.f.				

Propensity to howl versus bark was not significant in explaining our data distribution, but scan type (live animal versus excised larynx) did show a significant effect on both absolute and relative vocal fold length (table 3), with excised scans showing greater vocal fold lengths in both cases. This was unsurprising given that only wolf larynges were excised.

The rerun of the models, using only mesocephalic dogs and wolves as predictors, confirmed the previously mentioned results and showed mesocephalic dogs to have overall smaller larynges, as well as absolutely and relatively shorter vocal folds (centroid size: *F* = 5.49, *p* = 0.038; absolute vocal fold length: *F* = 10.53, *p* = 0.004; relative vocal fold length: *F* = 3.89, *p* = 0.064).

### 3.2. Larynx shape

**Figure 3.**
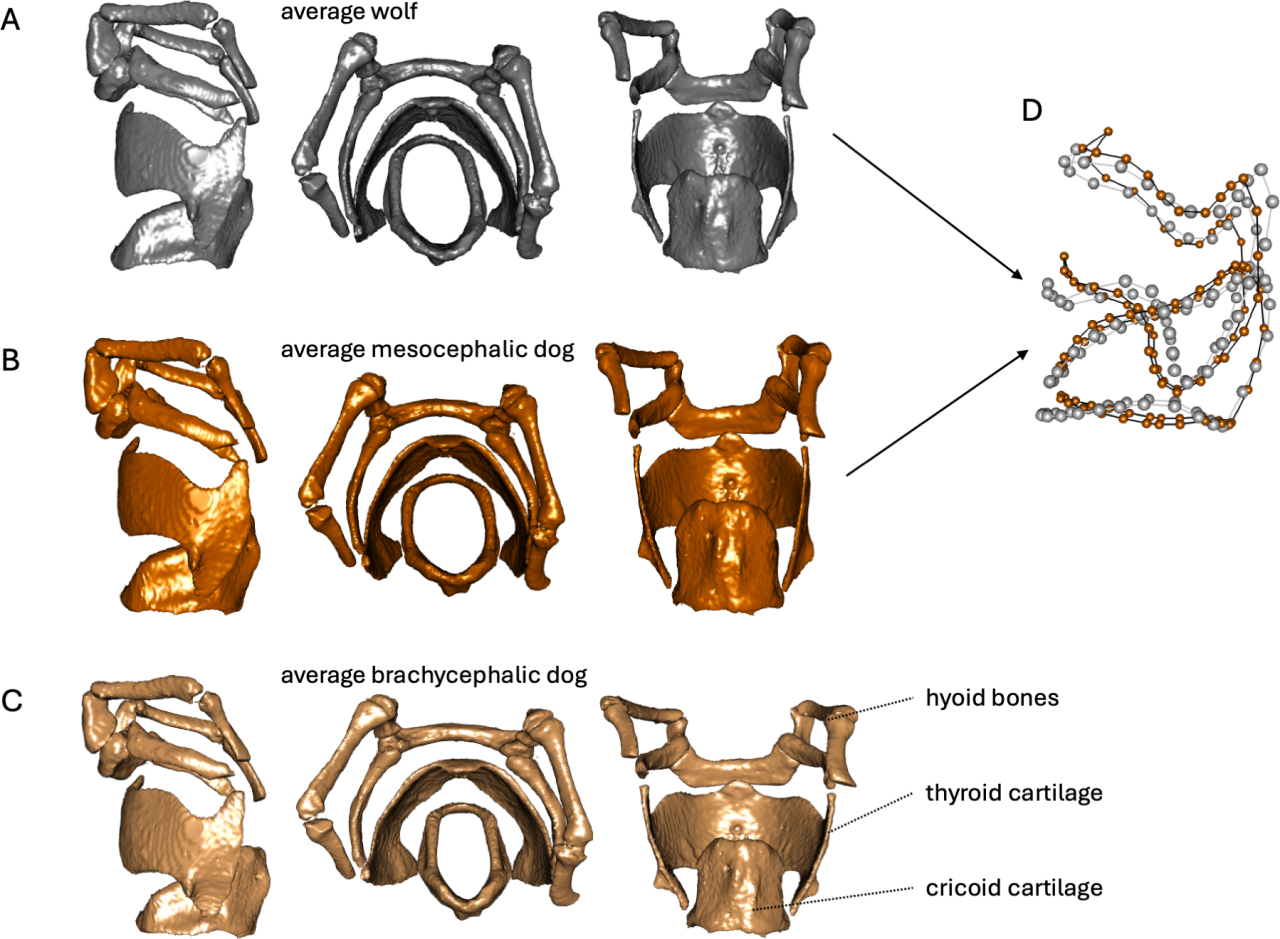
Lateral, cranial and dorsal views of three-dimensional morphs of each sample group. All the shape morphs derive from the three-dimensional reconstruction of CanLup002 (a wolf specimen), which was morphed into the average laryngeal shape of each sample group. Brachycephalic dogs are illustrated in light brown, mesocephalic dogs in dark brown and wolves in grey. (A) Morphs of the average wolf in grey. (B) Morphs of the average mesocephalic dog in dark brown. (C) Morphs of the average brachycephalic dog in light brown. (D) Three-dimensional illustration of the overlaid shape differences of the average wolf and mesocephalic dog larynx. This illustration is based on the PC 1 loadings’ minimum and maximum shape values of the mesocephalic dog wolf comparisons.

The full model for laryngeal shape was significantly better than the null model and also showed population identity to be a significant predictor (table 3). Scan type and propensity to howling versus barking had no impact on larynx shape. We were able to identify two main principal component (PC) dimensions capturing the shape change in our model. PC 1 captures 44.9% of the shape change and largely reflects the brachycephalic airway syndrome (figures 2D,E and 3). The component includes an overall flattening of the thyroid shield and a shortening and compression of the ventral-facing cricoid ring; PC 1 from here on is referred to as ‘BAS flattening’. This dimension shows an allometric relationship with centroid size (cor = 0.85, *p* = 9.433 × 10^−9^; figure 2E). This pattern is supported by our larynx centroid size results, with brachycephalic dogs having the smallest larynges and wolves the largest. PC 2 captures 10.3% of the shape variation and shows a decrease in interior cricoid diameter combined with straightening of dorsal thyroid horn curvature and upwards rotation of the central thyroid shield (figures 2D and 3); PC 2 is referred to as ‘domestic compression’ from here on. This dimension is not correlated with laryngeal centroid size.

The rerun of the models, using only mesocephalic dogs and wolves as predictors, confirmed the results of the complete data set (centroid size: *F* = 5.49, *p* = 0.038; shape: *F* = 3.25, *p* = 0.002; absolute VF: *F* = 10.56, *p* = 0.0044, relative VF: *F* = 3.898, *p* = 0.0639). The rerun of the PCA with only wolf and mesocephalic dog shape data indicated the first axis captured 27.9% of the shape variation among these two groups, and illustrated a shape change matching our description of ‘domestic compression’ (see the PC 1 scores in figure 2F). Furthermore, PC 1 is uncorrelated with larynx size (figure 2F), indicating that laryngeal shape differences between mesocephalic dogs and wolves are not driven by differences in overall larynx size.

## 4. Discussion

The results presented here are the first to explicitly test the predictions of the NCDS hypothesis regarding the larynx and provide support for the underlying mechanistic explanation of the NCDS [7,8]. Lesch & Fitch [16] proposed that, based on the mechanistic explanation of the NCDS hypothesis, all structures derived from the neural crest progenitor cells should be subject to size reductions and shape changes in domesticates, specifically pinpointing a previously undiscussed connection between the laryngeal cartilages and the neural crest.

Our results confirm that normal (mesocephalic) dogs’ larynges are reduced in overall size, have shortened vocal folds in absolute and relative terms and differ in larynx shape compared to those of wolves (defined here as ‘domestic compression’). When investigating anatomical changes to the larynx, we have to consider that the (mammalian) larynx is under intense pressure to retain its dual functionality of airway protection and voice production [18]. While the larynx has responded to various evolutionary pressures in impressive ways via size increases of the thyroid shield in howler monkeys [30] or more extreme anatomical rearrangements in baleen whales [31], mammalian larynges—limited by their dual functionality—do not stray too far from the ‘original mammalian’ blueprint [32,33]. When this functional duality of the organ is removed (due to the larynx no longer being used for vocalization), we see more extreme changes in laryngeal morphology, such as in toothed whales [34]. Hence, among closely related species that retain dual functionality, larynx shape differences are expected to be relatively subtle compared to mammal-wide variation. As made evident through the mesocephalic dog and wolf comparison, the effects of domestication on the larynx are nonetheless clearly visible, though both variant structures retain all traits characteristic of a canid larynx. In contrast, the larynx of brachycephalic dogs, due to severe artificial selection pressures for a short, broad face, deviates more strongly from this blueprint. Intense breed selection for heavily reduced snouts has impaired the functionality of the larynx in the process. All dogs, both mesocephalic and brachycephalic, have shorter absolute vocal fold lengths and smaller larynges than wolves.

Our results showed a clear pattern of smaller larynges and shorter vocal folds in mesocephalic dogs compared to wolves. We also found scan type (included in our models) to influence vocal fold length, but this is most likely to be an artefact of our sample: all scans of excised larynges came from wolves, which also tended to have longer vocal folds than the dogs. Aside from this artefact, we also found an allometric relationship between the laryngeal shape pattern, ‘BAS flattening’ and laryngeal size. However, this relationship proved to be driven by BAS flattening being prevalent in brachycephalic dogs, which also have the smallest larynges. Mesocephalic dogs cluster much closer to wolves with regard to BAS flattening, but nonetheless have on average smaller larynges than wolves. Overall, brachycephalic dogs seem to have undergone the same ‘domestic compression’ patterns as mesocephalic dogs, yet also present an additional layer of more drastic changes associated with pedigree breeding for reduced snouts.

Changes to laryngeal anatomy and organization become even more intriguing in light of extreme vocalizations. Basenjis, an ancient African dog breed, yodel but do not bark. Research on the laryngeal soft tissue anatomy has suggested that the distinct lack of barking is caused by anatomical changes in soft tissue organization [35]. The two females used in that study showed absent/reduced laryngeal saccules and shallow laryngeal recesses, potentially explaining their reduced ability to produce barks [35]. Inspired by the research on basenjis, we included propensity to barking or howling as predictors to laryngeal shape change in our study. Both wolves and huskies howl frequently compared to other dog breeds, but our results did not link the propensity to howling/barking to any anatomical changes within the cricoid or thyroid cartilage. There are multiple potential explanations for this. (i) Anatomically relevant changes connected to vocalization type might be more closely connected to soft tissues (as suggested with the basenjis) rather than cartilaginous structures. Research on the relevance of vocal tract soft tissues in sound modulation would further support that view [36]. (ii) Vocalization type might not be tied to any concrete anatomical structure at all, but could instead be driven by neurological/cognitive pathways. (iii) A combination of multiple factors contributes to the propensity and production of howls/barks across different canid sample groups.

The overall anatomical functionality of the cricoid and thyroid cartilages of the canid larynx do not seem to be compromised or altered in drastic ways in mesocephalic dogs, but the opposite is the case for brachycephalic dogs. Veterinary literature describes the brachycephalic airway (obstruction) syndrome (BAS) as a combination of upper respiratory symptoms caused by severe anatomical changes. Authors describe elongated soft palates, narrow nostrils, collapsible tracheas, soft tissue oedemas in the larynx and soft tissues, everted saccules, laryngeal collapse and decreased arytenoid cartilage stiffness caused by anatomical deficits and made worse by increased airway resistance and laboured breathing [37–40]. While BAS is well described and widely treated in veterinary medicine, a detailed description of the effect of brachycephaly on the laryngeal cartilage anatomy is lacking. We found brachycephalic dogs to suffer from a severe flattening of the thyroid cartilage and compression of the ventrally facing cricoid (which we referred to as ‘BAS flattening’). This finding is very much in line with the somewhat vague yet often described ‘laryngeal collapse’. Authors describe narrower, less rigid airways causing more laboured breathing, which in turn creates increased negative pressure in the airways, further compromising weakened laryngeal structures [41,42]. Our results concerning BAS flattening, with the thyroid cartilage caving inwards, potentially illustrates additional invasion of the airways. Flattening of the thyroid cartilage could cause additional laxity of the vocal folds, again increasing the airflow resistance and difficulty in breathing. In conjunction with the already reduced stiffness of the arytenoids in brachycephalic dogs the glottis would experience further decrease in airflow capacity [39].

Another interesting result in the context of BAS is the relatively high vocal fold to larynx size ratio in brachycephalic dogs, suggesting that vocal fold length is conserved to some extent. However, the severe shape change (i.e. flattening of the thyroid) and the reported changes in arytenoid stiffness in combination with very small larynges may lead to an overestimation of vocal fold length. However, the overall anatomical changes we describe in the laryngeal cartilage anatomy match behavioural and anatomical traits, and clinical symptoms described within the brachycephalic airway syndrome (BAS; [22,23,43]).

In conclusion, our results support the predictions of the NCDS hypothesis, namely that domesticates have reduced (laryngeal) structures compared to their wild counterparts. We demonstrate detailed anatomical changes regarding size reductions and shape changes in the larynges of mesocephalic dogs compared to those of wolves. Of course, we acknowledge that comparisons of the larynges of modern dogs and wolves only lets us imperfectly infer anatomical traits of early domesticated dogs and ancestral wolves. Our sample comprised adult captive wolves, various mesocephalic breeds of domestic dogs and a brachycephalic breed representative (French bulldogs). As illustrated by Balcarcel *et al*. [44] in a review of all available literature on domestication related brain size reductions, the particular sample used critically impacts the reported changes. We want to highlight that the sample presented here (for details, see table 1) was the result of multiple years of data collection with the goal of obtaining the most representative sample. Despite these efforts, we are aware that the comparisons might be impacted by effects of bottlenecking, inbreeding and genetic drift in dog and wolf populations [4,45]. However, because the larynx does not fossilize, modern comparisons are all that are available. Nonetheless we find mesocephalic dogs to have overall smaller larynges, shortened vocal folds and altered laryngeal cartilages, i.e. thyroid shield rotation/horn curvature straightening and cricoid diameter loss, which we defined as ‘domestic compression’. Our comparison of the larynges of dogs and wolves is a first test of the larynx-specific predictions of the NCDS and support the mechanistic explanation of the domestication syndrome by [7,8]. Aside from testing for effects of domestication on the larynx, we also investigated the anatomical changes to laryngeal anatomy caused by strong artificial selection pressures on airway-related structures (i.e. the BAS), approaching functional limits. This current study lays the foundation for comprehensively testing predictions of the NCDS hypothesis outside the classically mentioned ‘domestication syndrome’ traits, via detailed quantification of anatomical shape changes, and could easily be applied to other domesticated mammal species where representatives of the ancestral type remain available, such as cats, pigs, goats, sheep and/or guinea pigs.

## Data Availability

Data and code is accessible on Dryad [46].
